# Sirt1 deacetylates and stabilizes p62 to promote hepato-carcinogenesis

**DOI:** 10.1038/s41419-021-03666-z

**Published:** 2021-04-14

**Authors:** Lifeng Feng, Miaoqin Chen, Yiling Li, Muchun Li, Shiman Hu, Bingluo Zhou, Liyuan Zhu, Lei Yu, Qiyin Zhou, Linghui Tan, Huimin An, Xian Wang, Hongchuan Jin

**Affiliations:** 1 Laboratory of Cancer Biology, Key Lab of Biotherapy in Zhejiang Province, Cancer Institute of Zhejiang University, Sir Run Run Shaw hospital, School of Medicine, Cancer Center, Zhejiang University, Hangzhou, Zhejiang China; 2 Department of Pathology, Sir Run Run Shaw Hospital, Medical School of Zhejiang University, Hangzhou, China; 3 Department of Medical Oncology, Key lab of Biotherapy in Zhejiang, Sir Run Run Shaw Hospital, Medical School of Zhejiang University, Hangzhou, China

**Keywords:** Cancer, Post-translational modifications

## Abstract

p62/SQSTM1 is frequently up-regulated in many cancers including hepatocellular carcinoma. Highly expressed p62 promotes hepato-carcinogenesis by activating many signaling pathways including Nrf2, mTORC1, and NFκB signaling. However, the underlying mechanism for p62 up-regulation in hepatocellular carcinoma remains largely unclear. Herein, we confirmed that p62 was up-regulated in hepatocellular carcinoma and its higher expression was associated with shorter overall survival in patients. The knockdown of p62 in hepatocellular carcinoma cells decreased cell growth in vitro and in vivo. Intriguingly, p62 protein stability could be reduced by its acetylation at lysine 295, which was regulated by deacetylase Sirt1 and acetyltransferase GCN5. Acetylated p62 increased its association with the E3 ligase Keap1, which facilitated its poly-ubiquitination-dependent proteasomal degradation. Moreover, Sirt1 was up-regulated to deacetylate and stabilize p62 in hepatocellular carcinoma. Additionally, Hepatocyte Sirt1 conditional knockout mice developed much fewer liver tumors after Diethynitrosamine treatment, which could be reversed by the re-introduction of exogenous p62. Taken together, Sirt1 deacetylates p62 at lysine 295 to disturb Keap1-mediated p62 poly-ubiquitination, thus up-regulating p62 expression to promote hepato-carcinogenesis. Therefore, targeting Sirt1 or p62 is a reasonable strategy for the treatment of hepatocellular carcinoma.

## Introduction

Liver cancer is the fourth most common cause of cancer-related death and ranks sixth in terms of incidence worldwide^1^. Hepatocellular carcinoma (HCC) accounts for 85–90% of liver cancer cases. Surgical therapy is the first choice for patients at an early stage, but ~70% of them have tumor recurrence after 5 years^2^. So systemic therapies are essential for HCC patients. Despite great progress in treatment strategies, HCC still remains one of the most lethal malignancies, with a 5-year survival of 18%^3^. It is urgently needed to further investigate the mechanism of hepatocellular carcinogenesis for precision therapies. p62 (SQSTM1, sequestosome 1), an adapter protein in autophagy, plays a significant role in cellular homeostasis. In addition, it acts as a central hub to activate many signaling pathways including Nrf2, mTORC1 and NFκB signaling through its ability to interact with key signaling proteins such as Keap1, TRAF6, IKK2/β, etc^4^. p62 is frequently up-regulated in tumors, such as hepatocellular carcinoma (HCC) and breast cancer^5^. Transgenic overexpression of p62 could increase c-Myc production to stimulate the development of HCC via activating the mTORC1 signaling^6^, highlighting the relevance of p62 to HCC. However, how p62 was up-regulated in HCC remains largely undefined.

As the critical adapter protein in autophagy, p62 is degraded in the autolysosome together with its cargoes. Therefore, autophagy was recognized as the major mechanism to regulate p62 homeostasis.The chronic liver damage resulted from genetic inactivation of autophagy in liver parenchymal cells can be reversed by global *SQSTM1* gene ablation^7^. On the other hand, the depletion of Atg5 or Atg7, essential autophage-related genes, promoted liver tumorigenesis via p62 accumulation in HCC cells^8^. However, autophagy is often activated rather than inhibited in cancers cells to enable their survival under unfavorable circumstance such as hypoxia or nutrient shortage^9^, indicating that cancer-specific up-regulation of p62 is most likely autophagy-independent. For example, we have previously reported that transcription factor Yin Yang 1 (YY1) up-regulated p62 expression in breast cancer through the epigenetic silence of *MIR372* which targets p62 directly^5^. In addition, we have found Nrf2-dependent p62 transcription promotes tamoxifen-associated endometrial hyperplasia^10^.

Post-translational modifications such as phosphorylation, ubiquitination, acetylation, and methylation, play crucial roles in protein expression and function. It has been reported that mTORC1-mediated p62 phosphorylation at S349 could increase its affinity to Keap1, thus restraining Keap1-mediated ubiquitination and subsequent degradation of Nrf2. As a result, Nrf2 was over-activated to facilitate hepato-carcinogenesis^11^. Under oxidative stress, fructokinase A (KHK-A) could block p62 ubiquitination by phosphorylating it at S28, further enhancing its aggregation with Keap1 to activate Nrf2 in HCC cells^12^. Moreover, in neuronal cells, p62 can be ubiquitinated at K13 by the E3 ligase PARKIN, then subjected to proteasomal degradation^13^. Additionally, TRIM21 ubiquitylates p62 at K7 and suppresses protein sequestration to regulate redox homeostasis in liver^14^.

In this study, we found that type III deacetylase Sirtuin 1 (Sirt1) deacetylates p62 at K295, which suppresses Keap1 mediated ubiquitination-dependent degradation of p62 in HCC. Sirt1 expression was up-regulated in human HCC, while Hepatocyte-specific knockout of Sirt1 retarded hepato-carcinogenesis in mice via reducing p62 expression. Therefore, Sirt1-dependent p62 stabilization is critical for HCC development and targeting Sirt1 or p62 could be a promising intervention strategy for HCC.

## Materials and methods

### Cell cultures, reagents, and antibodies

Human liver cancer cell lines PLC/PRF/5, Huh7, and SK-Hep1 were all purchased from Cell Bank of the Typical Culture Preservation Committee, Chinese Academy of Sciences (Shanghai, China). HCCLM3 were purchased from the Liver Cancer Institute of Fudan University (Shanghai, China). PLC/PRF/5, Huh7, and HCCLM3 were cultured in DMEM medium (Invitrogen, Shanghai, China). All medium were supplemented with 10% FBS and 100 U/mL penicillin–streptomycin. PLC/PRF/5-shNC and PLC/PRF/5-shp62 cells were generated via infection of lentivirual containing shRNA (shNC or sh*SQSTM1*) and puromycin selection. All cell lines were authenticated by STR profiling and tested as mycoplasma-free.

The chemicals used in this study include: Trichostatin A (TSA) (Selleck, Shanghai, China, S1045; 1 μM, 12 h); Nicotinamide (NAM) (Selleck, S1899; 5 mM, 6 h); EX527 (Selleck, S1541; 25 μM, 16 h); chloroquine (CQ) (Sigma-Aldrich, Shanghai, China C6628; 25 μM, 16 h); MG132(Calbiochem, USA, 474790; 1 μM, 16 h); chlorhexidine (CHX) (Sigma-Aldrich, C7698,50 μg/mL).

The antibodies used were listed as: anti-p62 (MBL, Japan, PM045); anti-beta-actin (CST,USA, 8457); anti-Flag (Sigma-Aldrich, F1804-1); anti-acetyl Lysine (Abcam, USA, ab21623); anti-p53-Acetyl (CST, 2525s); anti-Sirt1 (Millipore, USA, 07–131); anti- mTOR-phospho (CST, 5536S); anti-Ub (Santa cruz, USA, sc-8017); anti-KEAP1 (Proteintech,Wuhan, China, 10503-2-AP); anti-Culin3 (CUL3) (CST, 10450); anti-Myc (CST, 2276); anti-GCN5 (CST, 3305); anti-SIRT1(Abcam, Ab32441); and anti-HA (Sigma-Aldrich, 11867423001).

### RNA extraction and quantitative real-time PCR

Total RNAs were isolated using the Trizole reagent (Invitrogen) and concentrations were quantified using NanoDrop 2000 (Wilmington, DE, USA), followed with DNase I digestion and reverse transcribed by random primers to generate cDNA templates strictly according to the manufacturer’s instructions (Thermo Fisher Scientific Inc., Shanghai, China). Quantitative real-time PCR was performed using SYBR Green Master Mix (CWBIO Biosciences, Beijing, China) and Light Cycler 480 II system (Roche, Shanghai, China). To determine relative gene expression, RNA integrity was normalized to internal control β-actin. All primer sequences used for PCR are listed in Supplemental Table 1.

### Plasmids, siRNAs, and transfection

The plasmids of Sirt1, GCN5, Tip60, and Keap1 were purchased from Sino Biological Inc. (Beijing, China). The full length of SQSTM1(p62) was used as previous reported^15^, and its various truncation segments were cloned to EX05-Flag vector. The QuickChange II Site-Directed Mutagenesis Kit (Applied Biosystem, CA, USA) was used to generate p62 site-directed mutants according to the manufacturer’s instruction. Flag-tagged p62 truncated mutants were generated by cloning the corresponding p62 cDNA fragments into EX05-Flag vector using XbaI and AgeI-HF sites. Flag-tagged p62-△LB was generated by Seamless cloning flowing the protocol of MultiS One Step Cloning Kit (Vazyme C113, Nanjing, China). All mutants were confirmed by sequencing. Plasmids were amplified and purified with the EndoFree Plasmid Maxi Kit (QIAGEN) and transfected into cells with X-tremeGENE HP DNA Transfection Reagent (Roche Applied Science, Shanghai, China). All primer sequences used were listed in Supplemental Table 2. And the schematic diagram of truncations was performed via the soft DOG2.0.

And siRNAs mentioned in this article were synthesized by Gene Pharma Company (Shanghai, China), and transfected into cells with Lipofectamine^TM^ RNAiMAX transfection reagent (Thermo Fisher Scientific, USA) at a final concentration of 20–50 nM. All siRNAs sequences used were listed in Supplemental Table 3.

### Immunoprecipitation and western blot

Cells with indicated treatment were lysed in Triton buffer (50 mM Tris-HCl, 150 mM NaCl, pH 7.4, 0.5–1% Triton-X-100) supplemented with protease inhibitor cocktail. The harvested cell lysates were quantitated by BCA protein assay kit (Bio-Rad Laboratories, Hercules, CA, USA), 1 mg cell lysate was mixed with antibodies and rotated at 4 °C for overnight followed by addition of protein A/G sepharose beads. Immuno-complex were washed, denatured, and subjected to western blot. Samples were resolved by SDS-PAGE, transferred to PVDF membrane and probed with the indicated primary antibodies, then washed with TBS-T (TBS with 0.1% of Tween-20) and incubated with suitable HRP-conjugated second antibodies (Jackson ImmunoResearch Inc., PA, USA), after that, the membranes were sent to autoradiograph with enhanced chemiluminescence (EMD Millipore, MA, USA) and pictures were processed with Amersham Imager 600 system (GE Healthcare Life Sciences, Shanghai, China).

### Mass spectrometry analysis

HEK293T cells were transfected with Flag-p62, after 48 h, cell lysates were immunoprecipitated with Anti-Flag M2 Affinity Gel (Sigma-Aldrich, A2220), the immunoprecipitated complex was resolved by SDS-PAGE and stained with coomassie blue, the probable size bands were retrieved and analyzed by mass spectrometry liquid chromatography (MS/LC).

### Glutathione-S-transferase pull down

Full length of p62 cDNA was cloned into pGEX-4T-1 vector. Glutathione-S-transferase (GST-p62) fusion protein was expressed in *E. coli* BL21, and purified with GSH-sepharase 4B beads. Full length of Sirt1 cDNA was cloned into pET28 vector. His-Sirt1 fusion protein was expressed in *E. coli* BL21, purified with Ni-NTA-Beads, and eluted via imidazole elution. Purified His-Sirt1 was mixed with GST beads bound by GST-p62 overnight at 4 °C. Protein–protein interaction was analyzed by western blot.

### In vivo ubiquitination assay

HEK293T cells were transfected with constructs encoding Flag-p62 or p62 K13/295 R/Q mutant, HA-His-UB, Myc-Keap1, along with empty vector. Forty-eight hours after transfection, cells were treated with MG132 (20 μM) for 6 h and then lysed with denatured buffer containing 6M guanidine as described previously^16^. Flag-p62-ploy-Ub was purified by Ni-NTA-beads pull down and detected by immunoblot using Anti-Flag antibody.

### Immunohistochemistry staining

Immunohistochemistry (IHC) analysis was performed in two cohorts of human liver tissues, one containing 183 cases, including 93 HCC tissues and 90 non-tumor liver tissues, the other containing 197 cases, including 100 HCC tissues and 97 non-tumor liver tissues. Briefly, anti-p62 antibody (1:10,000) and anti-Sirt1 antibody (1:1000) were incubated with tissue sections overnight at 4 °C, following antigen retrieval and BSA blocking. After the binding of HRP-conjugated secondary antibody, the slides were incubated with diaminobenzidine (DAB), followed by hematoxylin counterstaining. The IHC results were assessed by two independent pathologists. The IHC results were evaluated by two reviewers and scored 0–3 with respect to intensity and estimated percentage of positive tumor cells. The maximum score in this system is 3 and minimum is 0. The tumors were regarded as immunopositive when the IHC score (percentage positivity × staining intensity) of >1 was obtained and negative when the IHC score was ≤1.

### Human liver tissue specimens

In total, 12 pairs of fresh HCC samples for western blot were collected at the Sir Run Run Shaw hospital (Hangzhou, China), informed consent was obtained from all individuals. And the HCC cohort cases (*n* = 93) for IHC staining were obtained from Xinchao Company (Shanghai, China), informed consent was obtained from all individuals.

### Primary mouse embryo fibroblast isolation and infection

Isolation of primary mouse embryo fibroblasts (MEFs) followed protocols previously reported. Briefly, Sirt1 ^fl/fl^ C57BL/6J mice were hybridized. At the time of 12.5–13.5 postcoitum, the embryos were dissociated and then trypsinized to produce single-cell suspensions. MEFs were cultured in DMEM medium, 37 °C, 5% CO2 incubator.

Sirt1 ^fl/fl^ MEFs were infected with adenovirus containing GFP-Cre plasmids and the MOI is ~50. After more than 5 days, the protein and mRNA expressions were detected.

### Animal studies

For assaying tumor growth in the xenograft model, 6-week-old BALB/c athymic nude mice (nu/nu, male, *n* = 5 for each group) purchased from SPF Biotechnology (Beijing, China) were used with each experimental group. In all, 5 × 10^6^ PLC/PRF/5 shNC/shp62 stable cells were resuspended in a total volume of 0.1 ml 1x PBS and injected subcutaneously into flanks of mice. The growth of tumors was measured three times a week and tumor volume (TV) was calculated according to the equation: TV = (L × W^2^)/2. Three weeks after injection, mice were euthanized, and the tumor tissues were then peeled off and weighed.

Sirt1^fl/fl^ and Albumin-Cre C57BL/6J mice were purchased from Model Animal Research Center of Nanjing University (Nanjing, China); Sirt1^fl/fl^ mice were hybridized with Albumin-Cre mice, and the acquired Sirt1 CKO mice (Sirt1^fl/fl^ + Albumin-Cre+/+) were selected for experiments (male, *n* = 11), Sirt1^fl/fl^ + Albumin-Cre−/− mice were used as wild-type control (WT, male, *n* = 15). Four WT mice were feed from the begin to the end of the animal experiment as blank control (male, *n* = 5) To induce hepatocellular carcinogenesis, 100 mg/kg DEN (diethylnitrosamine, Simga-Aldrich, N0759)^17^ was i.p. injected into 4-weeks-old male mice, and 2 weeks later, 3 mg/kg TCPOBOP (Sigma-Aldrich, T1443) was i.p. injected into the mice every 2 weeks for eight times. Ten months after the DEN injection, mice were euthanized. The liver tissues were collected and divided, one half was immediately frozen in liquid nitrogen and stored at −80 °C until sent for western blot analysis, another half was fixed with 4% formaldehyde immediately and sent for HE staining. The numbers of liver tumor (diameter >1 mm) of each mice was counted and Student’s *t* test was performed for statistical analysis. For re-introduction of p62 in Sirt1 CKO mice (*n* = 5 for each group), adeno-associated virus (AAV)-8 adenovirus containing Flag-p62 or control plasimid constructed by Genechem company (Shanghai, China) were administered by slow intravenous injection in the tail vein every three months after DEN injection. No specific randomization method was used. All procedures involving animal handling were performed according to the institutional guidelines approved by the Animal Ethics Committee of the Capital Institute of Pediatrics.

### Statistical analysis

All data of relative mRNA expression, relative cell vitality, tumors numbers, and LW/BW were expressed as mean ± SD, and two- tailed Student’s *t*- test, was performed for statistical significance analysis. The chi-square test was performed for expression correlation analysis. Cox-regression analysis was performed for overall survival curve analysis. Exact sample size (*n*) is indicated in figure legend of each experiment. All the measurements were repeated at least three times with consistent trends. *p* value < 0.05 was considered as statistically significant. Data point is excluded if it deviates from mean with more than three standard deviations. No variation is estimated in the data of each group. All statistical analyses were performed using SPSS 22.0 software. Variance is similar between the groups that are being statistically compared. Investigators were not blinded to the group allocation during the experiment and when assessing the outcome in all experiments including animal experiments.

## Results

### High expression of p62 promotes hepato-carcinogenesis

**Fig. 1:**
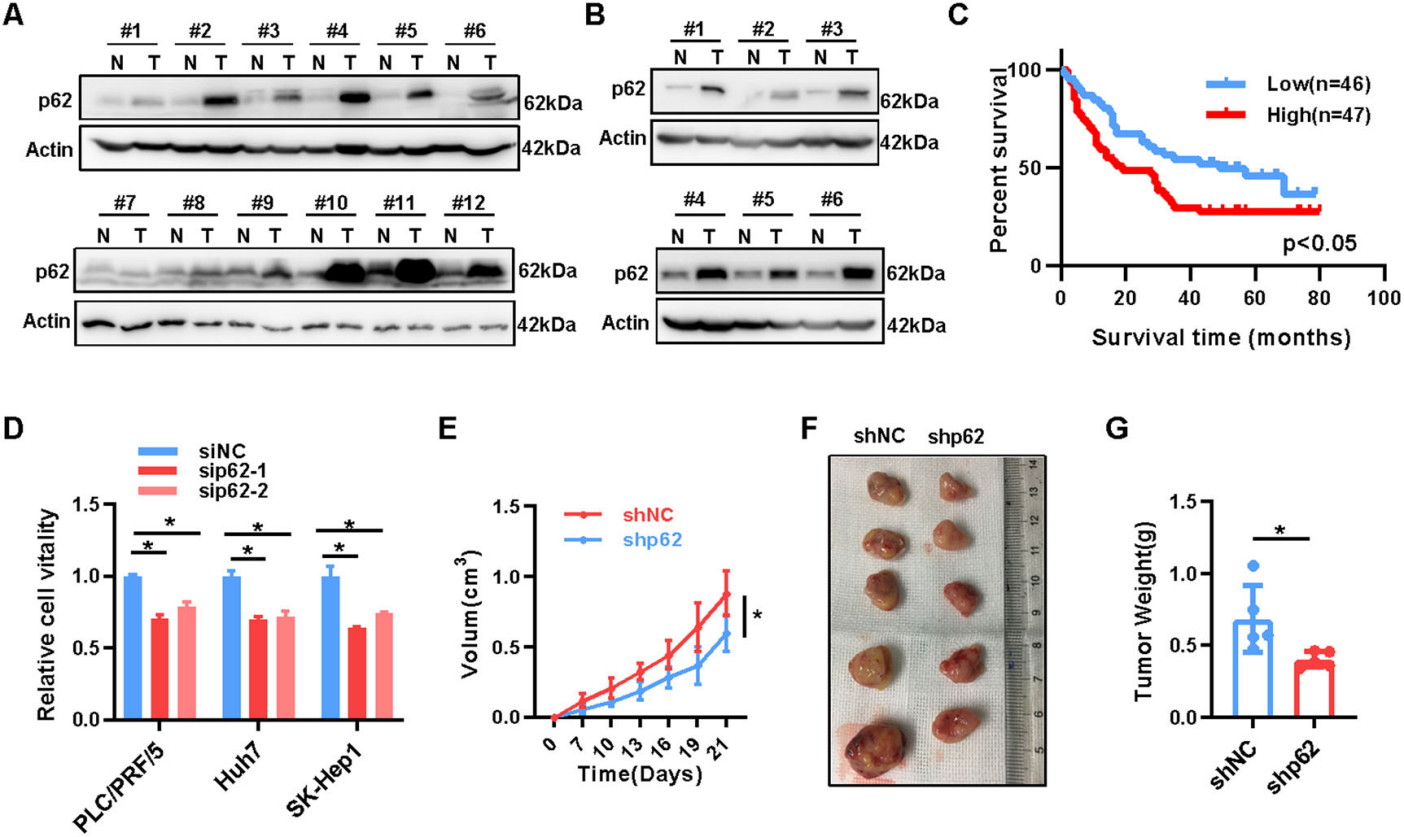
High expression of p62 promotes hepato-carcinogenesis. **A** Expression of p62 protein in 12 pairs of fresh HCC tissues and adjacent non-tumor tissues was analyzed by western blot. **B** p62 protein expression in 6 pairs of fresh liver cancer tissues and adjacent non-tumor tissues of DEN-induced mice liver cancer model was analyzed by western blot. **C** The impact of p62 protein expression on overall survival (OS) was analyzed by Kaplan–Meier survival curve analysis (*n*(Low) = 46, *n*(High) = 47, Cox-regression analysis, *p* < 0.05, Hazard Ration = 2.347, 95% CI of ratio 1.340–4.109). **D** Relative cell vitality of PLC/PRF/5, Huh7, and SK-Hep1 cells transient transfected with *SQSTM1*(p62) or negative control siRNAs was measured with MTS assay (*n* = 6, *T*-test, *p* < 0.05). **E** Growth curve, **F** representative image, and **G** Tumor weight of xenografts with stable p62-silenced (shp62) or scramble control (shNC) PLC/PRF/5 cells(*n* = 5, T-test, *p* < 0.05).

In order to clarify the relevance of p62 to HCC, we first analyzed p62 expression in adjacent non-tumor and tumor tissues of HCC patients, and observed high protein expression of p62 in 11 of 12 tumor tissues (Fig. 1A). We also found high expression of p62 in liver cancer tissues from DEN-induced mice (Fig. 1B). Additionally, higher p62 protein expression was associated with shorter overall survivals (OS) of HCC patients (Log-rank test, *p* < 0.05) based on data from a HCC cohort and TCPA dataset (https://tcpaportal.org; Fig. 1C and Supplemental Fig. 1A). Furthermore, knockdown of p62 significantly reduced the phosphorylation of mTORC1 (p-mTOR) in HCC cells and inhibited the viability of HCC cells in vitro (Fig. 1D and Supplemental 1B) and tumor growth in a xenograft mouse model (Fig. 1E–G and Supplemental Fig. 1E). In summary, our findings demonstrated that p62 acts as an oncogenic driver in HCC.

### Acetylation at lysine 295 reduces p62 stabilization

**Fig. 2:**
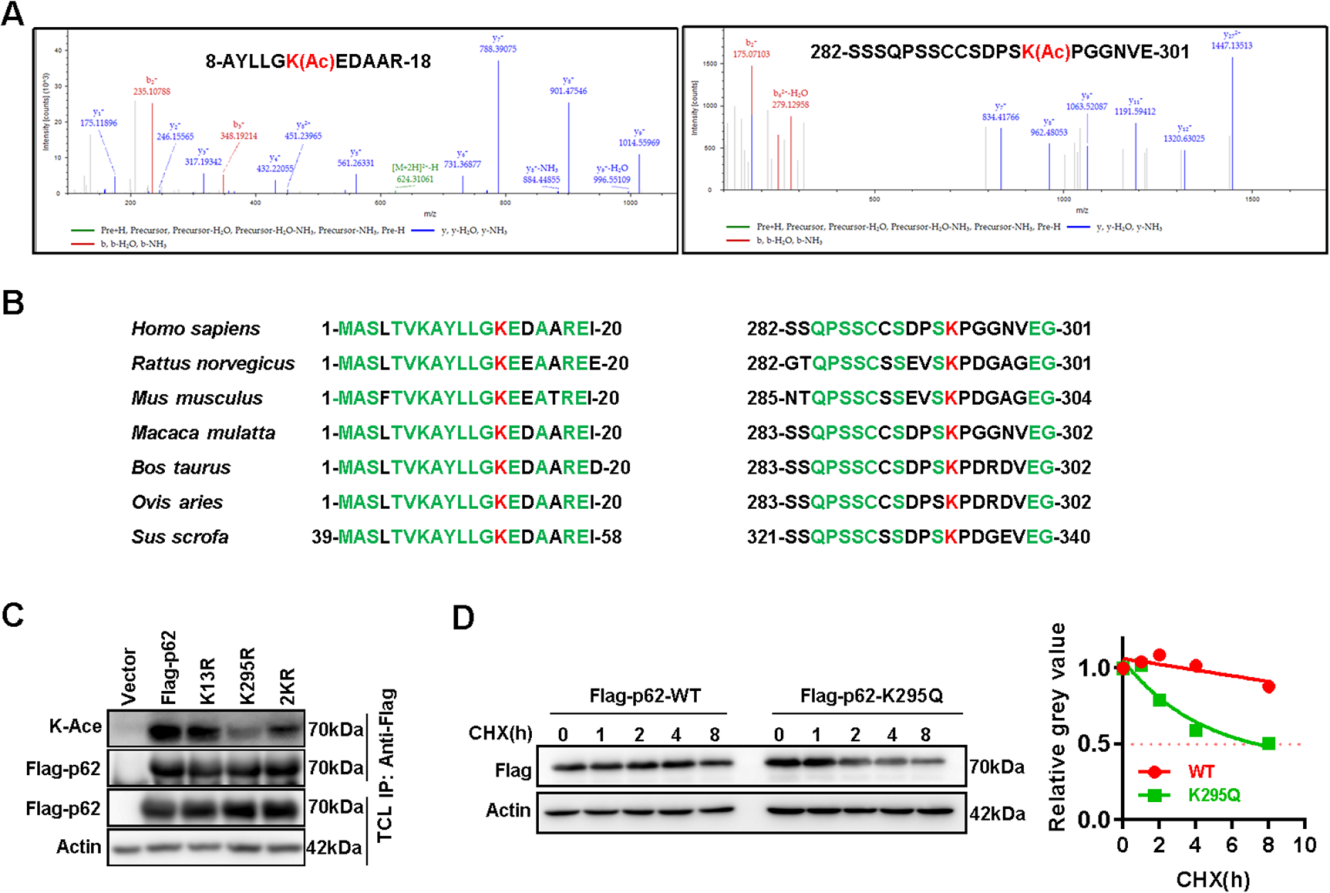
Acetylation at lysine 295 reduces p62 stabilization. **A** The acetylation site of Flag-p62 was analyzed by mass spectrometry (MS). The MS results showed that Lysine 13 (K13), 295 (K295) of p62 might be acetylated. **B** Conservation of p62 K13 and K295 in various species was aligned. **C** Acetylation of p62 wild type (WT) or p62 mutants (K13R or K295R) expressed in HEK293T cells were detected via western blot. **D** Wild type (WT) of p62 or p62(K295Q) mutants were overexpressed in HEK293T cells, followed cycloheximide (CHX) treatment with indicated time points, and the half-life of the WT and K295Q was detected via western blot, and the relative expression was measured by ImageJ software.

Next, we tried to explore the mechanism responsible for p62 up-regulation in HCC. Considering the importance of post-translational modification (PTM) in the regulation of protein expression and function, we performed mass spectrometry analysis to identify potential PTM sites in p62 protein. As a result, two novel acetylation sites, lysine 13 (K13) and lysine 295 (K295), were found to be lysine acetylated in exogenous p62 (Fig. 2A). Both of two sites are highly conserved among species (Fig. 2B). Acetylation-deficient mutants of K295 and K13 (lysine mutated to arginine, K to R) were then constructed respectively to verify their acetylation in HCC. Compared with wild-type p62 (Flag-p62/WT), p62-K295R (K295R) but not p62-K13R (K13R) showed markedly reduced acetylation (Fig. 2C), indicating the acetylation of p62 at lysine 295 site.

Interestingly, acetylation-mimicking mutant of p62 at lysine 295 site to glutamine (K295Q) displayed much shorter half-life than wild type p62 (Fig. 2D). Therefore, lysine acetylation at lysine 295 site decreases p62 stabilization.

### p62 is deacetylated by Sirt1 at K295

**Fig. 3:**
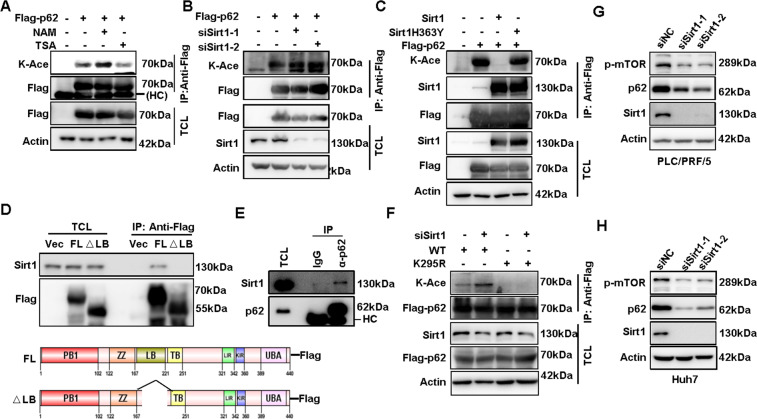
p62 is deacetylated by Sirt1 at K295. **A** Exogenous Flag-p62 in HEK293T cells treated with deacetylase inhibitors Nicotinamide (NAM, 5 mM, 6 h) or Trichostatin A (TSA, 1 μm, 12 h) was immunoprecipitated with anti-Flag, and p62 acetylation was analyzed using an anti-acetyl-Lysine (K-Ace) antibody via western blot. **B** Exogenous Flag-p62 in HEK293T cells after Sirt1 knockdown with siRNAs transfection was immunoprecipitated with anti-Flag, and the acetylation of p62 was analyzed using an anti-acetyl-Lysine (K-Ace) antibody via western blot. **C** Acetylation of exogenous Flag-p62 in HEK293T cells with or without co-overexpression of HA-Sirt1 or HA-Sirt1H363Y was detected by western blot. **D** Co-IP was performed to detect the interaction of full-length Flag-p62 or deletion (△LB (Lim-binding domain deletion, from amino acids 167–220)) with Sirt1 in HEK293T cells using anti-Flag, Empty vector was used as the negative control. **E** Co-IP was performed to detect the interaction of endogenous Sirt1 with p62 in PLC/PRF/5 cells using anti-p62, blank IgG was used as the negative control. **F** Acetylation of exogenous Flag-p62(WT) or mutant Flag-p62(K295R) in PLC/PRF/5 cells with or without Sirt1 knockdown and MG132 incubation was detected by western blot. **G**, **H** The protein expression of p62 and the phosphorylation of mTOR (p-mTOR) in PLC/PRF/5, Huh7 and SK-Hep1 cells with or without Sirt1 knockdown was analyzed by western blot.

To identify the deacetylases regulating p62 acetylation, two different deacetylases inhibitors were used separately to observe their effects on p62 acetylation, including type I and II HDAC family inhibitor trichostatin A (TSA) and type III HDAC family (Sirtuins) inhibitor Nicotinamide (NAM)^18^. We found that NAM rather than TSA could increase p62 acetylation, suggesting the involvement of Sirtuins in the regulation of p62 acetylation (Fig. 3A and Supplemental Fig. 2A). Indeed, EX527, a specific Sirtuin 1 (Sirt1) inhibitor, elevated the acetylation of p62 (Supplemental Fig. 2B). Similarly, knockdown of Sirt1 increased p62 acetylation (Fig. 3B and Supplemental Fig. 2C). On the other hand, overexpression of wild type Sirt1 but not the deacetylase-dead H363Y mutant^19^ eliminated p62 acetylation (Fig. 3C).

Moreover, the direct interaction of Sirt1 and p62 was verified by immunopreciptation (Supplemental Fig. 3A), as well as GST-pull down analysis with purified recombinant GST-p62 and His-Sirt1 (Supplemental Fig. 3B). To map the region responsible for the interaction of p62 with Sirt1, different truncated or deleted forms of Flag-p62 were built and examined the interaction with Sirt1 (Supplemental Fig. 3C, D). We found that Sirt1 is co-precipitated with most of p62 forms except the mutant with a deletion of amino acids 167–220 (p62-△LB), which are located at Lim-binding (LB) domain (Fig. 3D), indicating that LB domain is critical for the interaction of p62 with Sirt1. Endogenous Sirt1 was consistently found to be able to interact with p62 in HCC cells (Fig. 3E). Next, we want to know whether Sirt1 suppresses p62 acetylation at K295. As expected, knockdown of Sirt1 could evidently increase the acetylation of wild type p62 (WT) but not the K295R mutant in HCC cells (Fig. 3F). In addition, acetylation-mimicking mutant of p62 at lysine 295 (K295Q) displayed much shorter half-life than wild-type p62 in HCC cells (Supplemental Fig. 2E). As a result, Sirt1 knockdown greatly reduced p62 protein expression and the phosphorylation of mTOR (p-mTOR) in HCC cells (Fig. 3G, H and Supplemental Fig. 2D). Taken together, these results suggested that Sirt1 directly deacetylates p62 at K295, which might be important to stabilize p62 protein expression.

### Sirt1 inhibits GCN5-mediated p62 acetylation

**Fig. 4:**
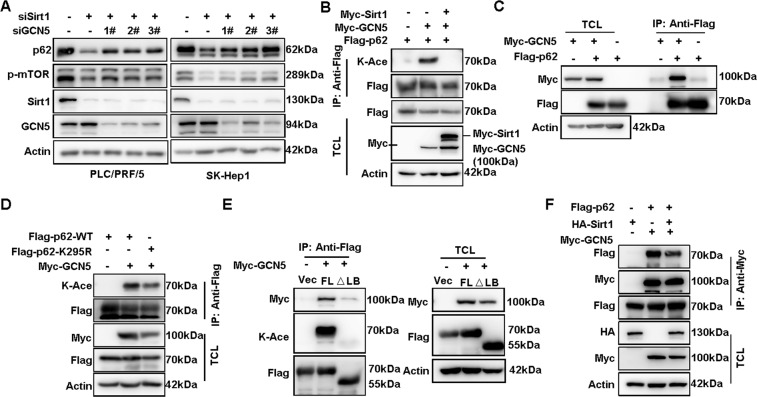
Sirt1 inhibits GCN5-mediated p62 acetylation. **A** The expression of p62 protein and the phosphorylation of mTORC1 in PLC/PRF/5 and SK-Hep1 cells with GCN5 siRNAs and Sirt1 siRNA co-transfection were analyzed by western blot. **B** Acetylation of Flag-p62 in HEK293T cells co-transfected with Myc-GCN5 and Myc-Sirt1 or empty vector were analyzed by anti-Flag immunoprecipitation, and probed with anti-K-ace. **C** Co-IP was performed to detect the interaction of Flag-p62 with Myc-GCN5 in HEK293T cells with anti-Flag. **D** Acetylation of the mutants of Flag-p62, WT, or K295R in HEK293T cells co-transfected with Myc-GCN5 were analyzed by anti-Flag immunoprecipitation, and probed with anti-K-ace. **E** Co-IP was performed to detect the interaction of Flag-p62 or Flag-p62 △LB with Myc-GCN5 in HEK293T cells. Flag-p62 or Flag-p62△LB and Myc-GCN5 co-transfection were analyzed with anti-Flag co-IP. And the acetylation of Flag-p62 or Flag-p62 △LB Flag-p62 in HEK293T with Myc-GCN5 overexpression was probed with anti-K-ace. **F** Co-IP was performed to detect the interaction of Flag-p62 and Myc-GCN5 with or without overexpression of HA-Sirt1 with anti-Myc.

To further identify the acetyltransferase of p62, we examined the potential relevance of the most common acetyltransferases to p62 protein level^20^. Among the acetyltransferases, only GCN5 seems to affect p62 expression since its knockdown led to a dominant increasing of p62 protein level after Sirt1 knockdown (Supplemental Fig. 4A–E and Fig. 4A). In addition, the effect of GCN5 to acetylate p62 was abrogated in the presence of Sirt1 overexpression (Fig. 4B). Moreover, the acetylation of K295R was much weaker in GCN5 over-expressed cells than WT cells (Fig. 4D), confirming the acetylation of lysine 295 by GCN5. Additionally, GCN5 could interact with p62 (Fig. 4C), and the LB domain of p62 was responsible for the interaction (Fig. 4E and Supplementary Fig. 4F). LB domain is also the dominant region for p62 to interact with Sirt1, so we inferred that physical occupation of the LB domain by Sirt1 would affect the interaction of GCN5 and p62. Indeed, over-expressed Sirt1 could impair the interaction of GCN5 and p62 (Fig. 4F). In summary, GCN5 could promote p62 acetylation at K295, which could be disrupted by over-expressed Sirt1.

### Sirt1 stabilizes p62 via inhibiting its acetylation

**Fig. 5:**
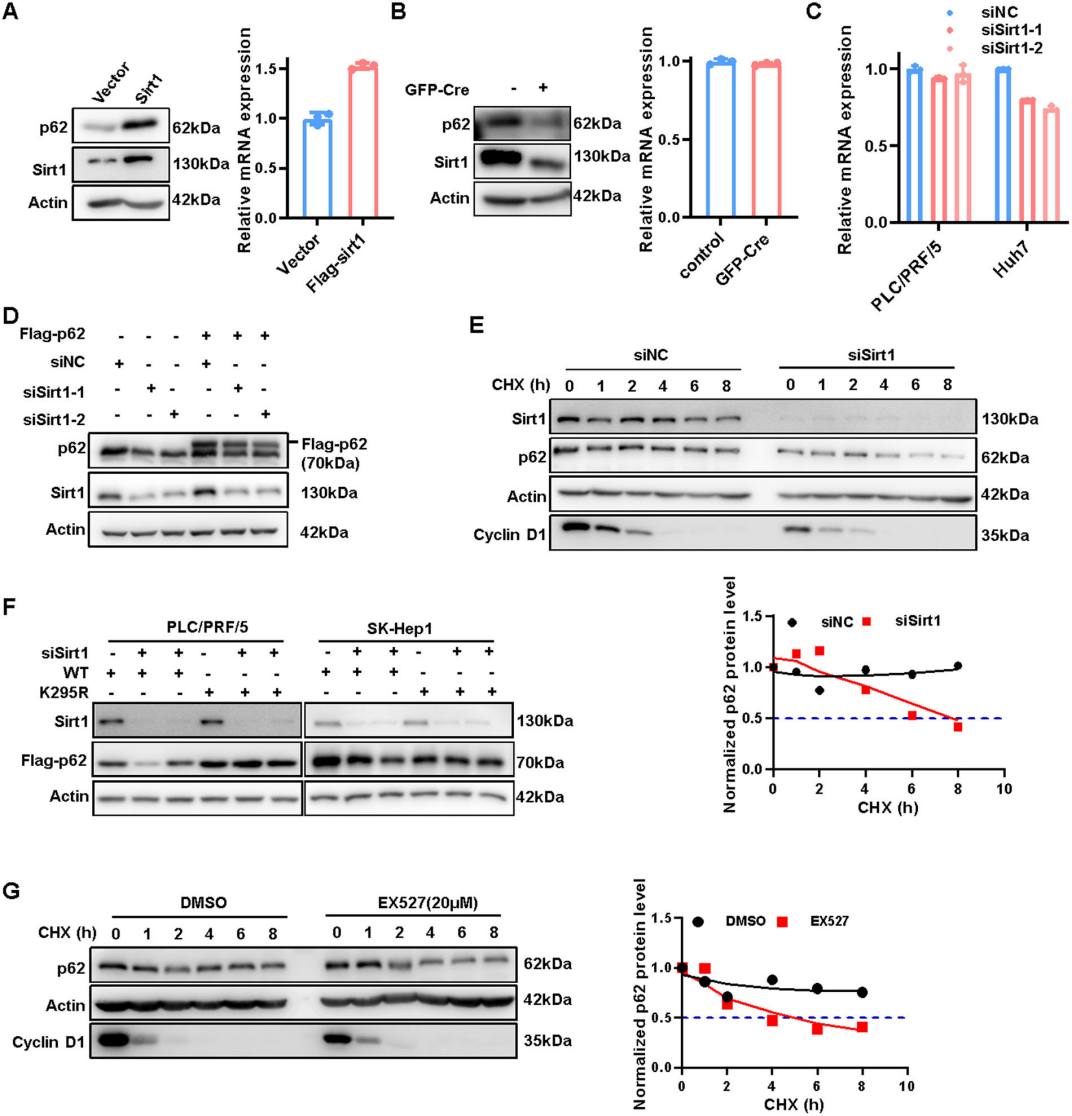
Sirt1 stabilizes p62 via inhibiting its acetylation. **A** The protein and mRNA expression of p62 in HCCLM3 cells with or without Sirt1 overexpression was analyzed by western blot or RT-PCR. **B** The protein and mRNA expression of p62 in of Sirt1^fl/fl^ MEF cells with or without GFP-Cre adenovirus infection were analyzed by western blot or RT-PCR. **C** The mRNA expression of p62 in PLC/PRF/5 and Huh7 cells with or without Sirt1 knowdown via siRNAs was analyzed by RT-PCR. **D** Exogenous Flag-p62 protein expression in PLC/PRF/5 cells with or without Sirt1 knockdown were analyzed by western blot. **E** The half-life of p62 in PLC/PRF/5 with or without Sirt1 knowdown via siRNAs was analyzed by western blot and the half-life of p62 in PLC/PRF/5 with or without Sirt1 knowdown via siRNAs was determined by ImageJ software. **F** Exogenous Flag-p62(WT) or p62 mutants (K295R) protein expression in PLC/PRF/5 and SK-Hep1 cells with or without Sirt1 knockdown were analyzed by western blot. **G** The half-life of p62 in PLC/PRF/5 with or without Sirt1 inhibitor (EX527, 20 μM, 24 h) treatment was analyzed by western blot and the half-life of p62 in PLC/PRF/5 with or without Sirt1 inhibitoor treatment was determined by ImageJ software.

Combining above results that lysine acetylation at K295 site decreases p62 stabilization and that Sirt1 eliminates p62 acetylation, we inferred that Sirt1 might be able to stabilize p62 via inhibiting its acetylation. Consistent with our expectation, overexpression of Sirt1 up-regulated p62 protein expression but not its mRNA level (Fig. 5A), while Sirt1 depletion led to downregulation of endogenous (Figs. 5B and 3G, H) and exogenous p62 protein (Fig. 5D and Supplementary Fig. 5A) with the mRNA expression unaffected (Fig. 5B, C). And the downregulation of p62 protein in the presence of protein synthesis inhibitor cycloheximide (CHX) was accelerated in HCC cell lines after Sirt1 knockdown (Fig. 5E). The results taken together supported that Sirt1 regulates p62 expression at the protein stability level rather than affecting the transcription process or mRNA stability.

We further explored whether the regulation of Sirt1 to p62 protein level was mediated through deacetylation at lysine 295 site. Indeed, Sirt1 knockdown decreased the expression of p62-WT but not p62-K295R expression in HCC cells (Fig. 5F). Consistently, inhibition of Sirt1 activity greatly reduced the half-life of endogenous p62 (Fig. 5G). In contrast to deacetylase-dead H363Y mutant (Sirt1-H363Y), wild-type Sirt1 (Sirt1-WT) could increase the protein expression of exogenous p62-WT (Supplementary Fig. 5B). However, the expression of K295R/Q mutant but not K13R/Q mutant, was not affected by Sirt1-WT compared to Sirt1-H363Y (Supplementary Fig. 5B). In summary, Sirt1 stabilizes p62 through deacetylating p62 at K295 in HCC cells.

### Sirt1 suppresses Keap1 mediated ubiquitination of p62

**Fig. 6:**
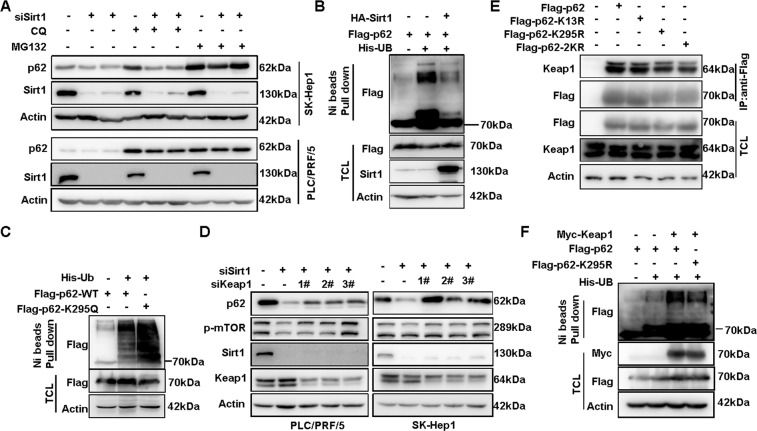
Sirt1 suppresses Keap1-mediated ubiquitination of p62. **A** p62 protein level in PLC/PRF/5 and SK-Hep1 cells treated with CQ(50 μM, 16 h) or MG132(5 μM, 16 h) after Sirt1 knockdown were detected by western blot. **B** Ni-NTA beads pull down was applied for ubiquitylation analysis of Flag-p62 in HEK293T cells co-transfected with Flag-p62, HA-sirt1 and His-ubiquitin (His-Ub) or empty vector, and anti-Flag antibody was used to detect ubiquitinated p62. **C** Ubiquitylation of WT or K295Q in HEK293T cells co-transfected with His-Ub or empty vector were analyzed by Ni-NTA beads pull down. **D** p62 protein level and the phosphorylation of mTORC1 in PLC/PRF/5 and SK-Hep1 cells with Keap1 siRNAs and Sirt1 siRNA co-transfection were analyzed by western blot. **E** Co-IP was performed to detect the interaction of WT or K295R with Keap1 in 293T cells by anti-Flag. **F** Ubiquitylation of Flag-p62 WT or K295R in HEK293T cells co-transfected with Keap1 and His-Ub or empty vector were analyzed by Ni-NTA beads pull down.

In an effort to explore the mechanism of p62 stabilization by Sirt1, we found that treatment with proteasome inhibitor MG132 other than lysosome inhibitor CQ could rescue the decrease of p62 protein after Sirt1 knockdown (Fig. 6A), indicating that acetylated p62 might undergo ubiquitination-dependent degradation. Indeed, ubiquitination of p62 was increased after knockdown of Sirt1 in HCC cells (Supplementary Fig. 6A). And, the in vivo ubiquitination assay showed that p62 ubiquitination was reduced upon Sirt1 overexpression (Fig. 6B). Furthermore, compared to p62-WT, p62-K295Q had more ubiquitination (Fig. 6C), while there was less ubiquitination of acetylation-deficient mutant K295R (Supplemental Figure 6B), highlighting that acetylated p62 undergoes ubiquitination-dependent degradation.

To identify the E3 ligase responsible for the ubiquitination of acetylated p62, we screened previously identified E3 ligases of p62, including Keap1/Culin-3^21^, Trim21^14^, RNF26^22^, and PARKIN^13^. Knockdown of Keap1 or Culin-3 but not the other E3 ligases could rescue the reduced p62 protein expression after knockdown of Sirt1 (Fig. 6E and Supplemental Fig. 6C–G). In the presence of over-expressed Keap1, a remarked ubiquitination of p62-WT was observed, compared to acetylation-deficient mutant K295R (Fig. 6E). In addition, the interaction between Keap1 and p62 was impaired in K295R mutant (Fig. 6F), and enhanced after Sirt1 knockdown (Supplemental Fig. 6H), confirming the importance of K295 acetylation for Keap1 interaction and subsequent ubiquitination-dependent degradation of p62. Taken together, these results suggested that Sirt1 deacetylated p62 at K295 to disrupt its interaction with Keap1, thus preventing its ubiquitination-dependent degradation.

### Sirt1 promotes hepatocellular carcinogenesis via up-regulating p62 expression

**Fig. 7:**
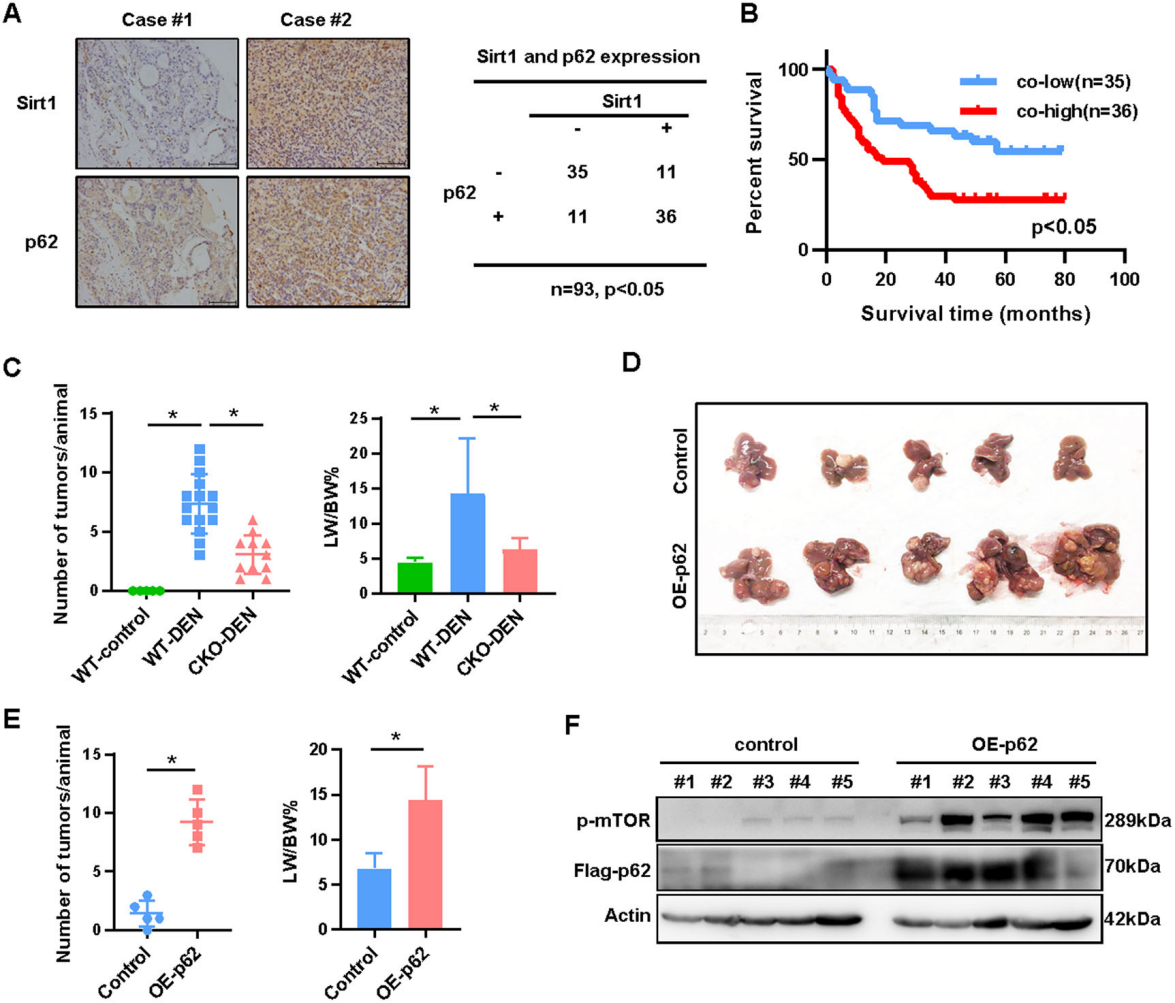
Sirt1 promotes hepatocellular carcinogenesis via up-regulating p62 expression. **A** Represented photos of p62 and Sirt1 expression in HCC tissues were shown, cases were grouped based on median expression and the correlation was analyzed (*n* = 93, *χ*^2^ test, *p* < 0.05 represents statistically significant). **B** Patients were grouped as above in **A**, and the overall survival of co-high (both Sirt1 and p62 high expressed) and co-low (both Sirt1 and p62 low expressed) groups of patients was analyzed via Kaplan–Meier survival curve analysis (*n* = 71, Cox-regression analysis, *p* < 0.05, Hazard Ration = 2.347, 95% CI of ratio 1.340–4.109). **C** The average tumor numbers (>1 mm) per mouse and the average liver weight/body weight ratio of three groups (wild-type mice without DEN treatment (WT-Control, *n* = 5), DEN-treated wild-type mice (Sirt1^fl/fl^, Alb-cre^−/−^, WT-DEN, *n* = 15) and DEN treated Sirt1 hepatocyte-specific knockout mice (Sirt1^fl/fl^, Alb-cre^+/+^, CKO-DEN, *n* = 11) were showed respectively. **D** The representative liver images, **E** the average tumor numbers (>1 mm) per mouse and the average liver weight/body weight ratio in CKO mice with p62 re-introduced (*n* = 5) or not (*n* = 5) were showed respectively. **F** The expressions of Sirt1, Flag-p62, and p62 downstream targets, p-mTOR were detected by western blot in liver tumors.

In line with previous reports, we confirmed higher expression of Sirt1 in human HCC samples and DEN-induced mice liver cancers compared to normal liver tissues (Supplemental Fig. 7A, B). Patients with higher Sirt1 expression in tumor tissues exhibited significantly shorter overall survival (Supplemental Fig. 7C). Consistent with our finding, the expression of Sirt1 was significantly correlated with p62 expression (Fig. 7A; *χ*^2^ test, *p* < 0.05). Furthermore, among 93 human HCC cases, 36 cases of co-high expression of p62 and Sirt1 exhibited significantly shorter overall survival than 35 cases of co-low expression (Fig. 7B; *p* < 0.05), demonstrating the relevance of Sirt1-p62 axis to HCC.

Additionally, Sirt1 knockdown significantly suppressed cell viability (Supplemental Fig. 7D), whereas Sirt1 overexpression promoted viability in HCC cell lines (Supplemental Fig. 7E). In DEN-induced mice model, the mice with hepatocyte-conditional knockout (CKO) of Sirt1 developed a less burden of liver tumors compared to wild-type (WT) mice (Fig. 7C and Supplemental Fig. 7F). Moreover, the level of p62 protein and p-mTOR was lower in liver tumors of Sirt1 CKO mice than WT mice (Supplemental Fig. 7G). To investigate whether p62 is indeed required for Sirt1-promoted HCC in vivo, we hydrodynamically injected Sirt1 CKO mice with adeno-associated virus (AAV) 8 carrying Flag-p62-encoding vector to introduce exogenous p62 into liver. As a result, overexpression of p62 in liver reversed the decline of tumor burden in Sirt1 CKO mice (Fig. 7D–F). Taken together, these data indicated that Sirt1 promotes hepatocellular carcinogenesis through stabilizing p62 protein.

## Discussion

**Fig. 8:**
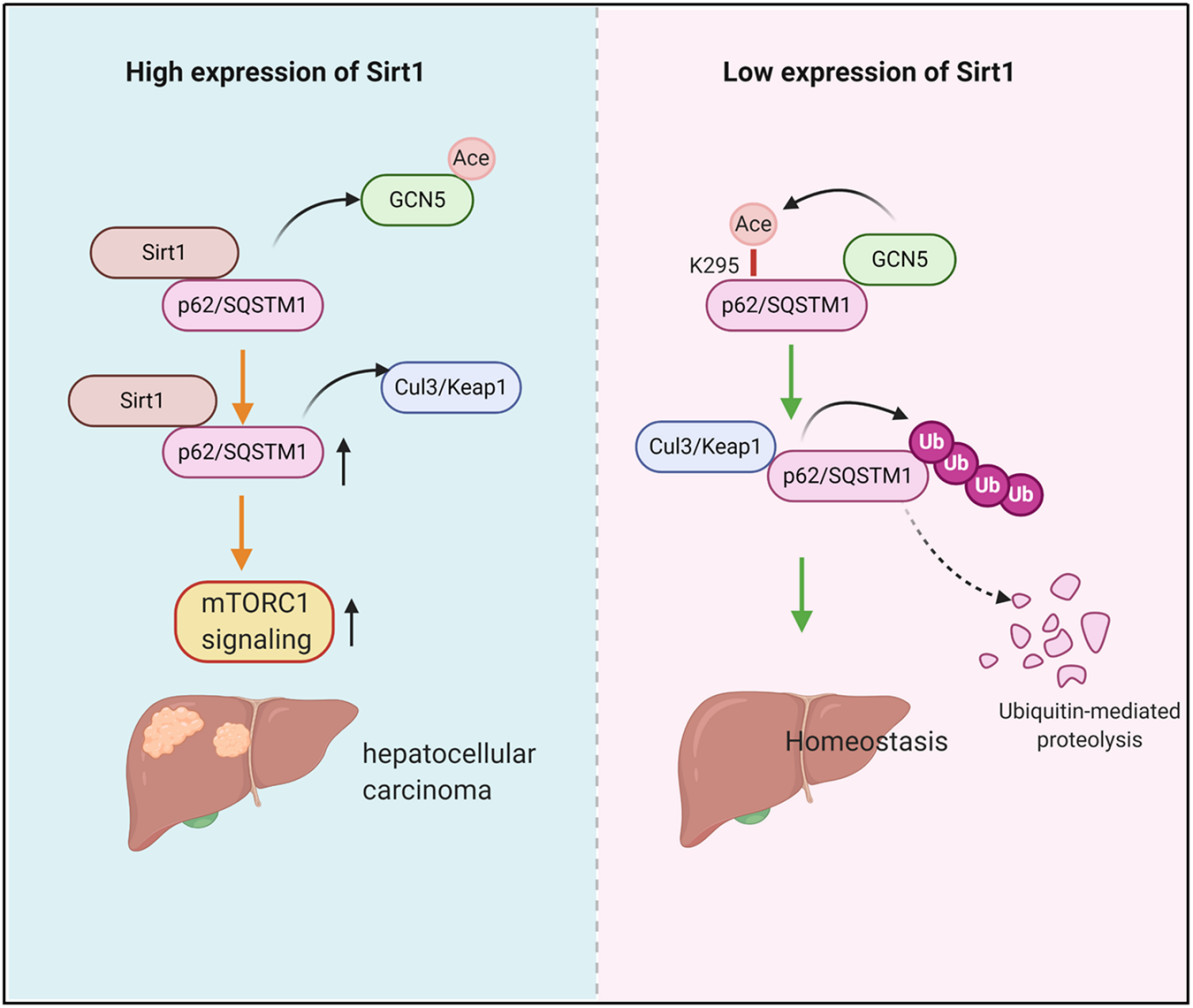
The model of the regulation mechanism of Sirt1-p62 axis. Type III deacetylase Sirt1 interacted with p62 to decrease its acetylation mediated via GCN5 at K295 and disturbed Keap1 mediated ubiquitination-dependent degradation of p62. Thus, Sirt1 up-regulation resulted into p62 accumulation, and contributed to hepatocellular carcinogenesis.

p62 has been recognized as a new oncoprotein in various cancers including HCC, with the potential to activate multiple oncogenic signaling pathways such as mTOR and Nrf2. High p62 expression, which could be resulted from the blockage of autophagy, was associated with a poor clinical prognosis. However, autophagy was frequently activated to enable cellular survival of tumor cells under unfavorable circumstance such as hypoxia and nutrient deficiency, indicating alternative mechanisms for the up-regulation of p62 during cancer development. Here we reported that type III deacetylase Sirt1 interacted with p62 to decrease its acetylation at K295 and disturbed Keap1 mediated ubiquitination-dependent degradation of p62. Thus, Sirt1 up-regulation resulted into p62 accumulation, activation of mTORC1signaling, and contributed to hepatocellular carcinogenesis (Fig. 8).

Previous study showed that p62 modulates selective autophagy by binding to LC3 via its LIR motif and accumulating polyubiquitinated proteins or organelles via its C terminal UBA motif^23^. Interestingly, p62 itself is also a selective substrate of autophagy, making autophagy an important mechanism to regulate p62 expression. Indeed, previous reports showed up-regulated p62 expression in autophagy-deficient HCC cell lines, and p62 ablation inhibited cell proliferation^7^. Moreover, p62 depletion in autophagy deficient mice liver attenuated hepatocellular carcinogenesis^24^. On the other hand, our recent study have found that EGFR TKIs (tyrosine kinase inhibitors for epidermal growth factor receptor) impaired lysosome function and prevented autolysosomal degradation of p62, which conferred NSCLC acquired resistance to EGFR TKIs^25^. However, mutations of autophagy-related genes such as ATG5 and ATG7 are rare and autophagy can be activated in cancer cells under various stress conditions, indicating autophagy-independent regulation implicated in p62 expression.

In fact, it has been reported that many oncogenic signalings could up-regulate p62 expression in various types of cancers including pancreatic ductal adenocarcinoma, breast cancer and so on. For examples, activated signaling pathways such as NFκB, Nrf2 or Ras/MAPK promoted p62 transcription^10,26^, and p62 mRNA-targeting miR-372 was epigenetically down-regulated to stimulate p62 mRNA translation^27^. Additionally, protein post-translational modifications are implicated in the expression and function of p62. CDK1 or p38δ stimulates p62 T269/T272 phosphorylation to enhance its interaction with TRAF6, thus activating mTORC1-dependent signaling pathway^28,29^. Meanwhile, mTORC1 mediated p62 S349 phosphorylation remarkably increases the binding affinity of p62 to E3 ligase Keap1, thereby sequestering Keap1 from Nrf2 to suppress the ubiquitination-dependent degradation of p62^30^. Casein kinase 2 (CK2) and TBK1 phosphorylate S403 of p62 in its UBA domain to enhance the affinity between UBA and polyubiquitinated chain, which promotes p62 separation and selective autophagy^31,32^. In addition to phosphorylation, ubiquitination is also involved in the regulation of p62 function^31^. For example, RING finger domain-containing ubiquitin E3 ligase TRIM21 ubiquitylates p62 at K7 via K-63 linkage to abrogate p62 oligomerization and sequestration of proteins including Keap1, eventually attenuating anti-oxidant response under oxidant stress^14^. On the other hand, Keap1/Cullin3-mediated ubiquitination at K420 facilitates the formation and degradation of ubiquitinated aggregates^21^. Additionally, Parkin directly interacts with and ubiquitinates p62 at K13 to promote proteasomal degradation of p62. And dysregulation of parkin/p62 axis may contribute to the onset of PD pathogenesis^13^.

In the current study, we identified that like the well-recognized phosphorylation and ubiquitination modifications, acetylation is also implicated in the regulation of p62 protein expression. p62 acetylation at K295 enhances its binding affinity with Keap1, further facilitating its ubiquitination-dependent proteasomal degradation (Fig. 6F). During hepatocellular carcinogenesis, Sirt1 expression was up-regulated to suppress GCN5-mediated p62 acetylation at K295 and stabilize p62 protein via retarding Keap1 dependent ubiquitination and subsequent proteasomal degradation. Recently, it was found that Tip60 writes and HDAC6 erases acetylation of p62 at K420 and K435 in its UBA domain, which enhances poly-ubiquitylated chains binding by disrupting UBA dimerization and increases UBA-ubiquitin affinity^20^. However, we found that knockdown of Tip60 could not reverse p62 downregulation resulted from Sirt1 knockdown (Supplemental Fig. 4A). In addition, GCN5 could stimulate more pronounced p62 acetylation than Tip60 (Supplemental Fig. 4G). Therefore, GCN5 might be the major acetyltransferase for p62, at least in HCC cells. Certainly, we cannot exclude other sites to be acetylated in HCC cells. Under GCN5 overexpression, acetylation of p62-K295R is remarkably reduced, but not disappeared. Interestingly, we found that LB domain of p62 is responsible for its interaction with both Sirt1 and GCN5, and over-expressed Sirt1 could compete with GCN5 for p62 interaction (Figs. 3D and 4E, F). These observations indicated that Sirt1 can not only actively deacetylate p62, but also prevent its acetylation by competing for GCN5 binding. Therefore, Sirt1 could be an ideal target for HCC treatment.

As the most extensively studied sirtuins that deacelate histones and non-histone proteins such as p53, FOXO, and c-Myc, Sirt1 regulates diverse biological processes such as DNA repair, metabolism, and cell growth^33^. However, it seems that the role of Sirt1 differs in different caner types. In breast, lung, and liver cancers, Sirt1 functions as an oncogene to promote cancer cell proliferation. In contrast, Sirt1 inhibits cell growth as a tumor suppressor in prostate cancer and glioblastoma^34^. In HCC, increased expression of Sirt1 promotes hepatocellular carcinogenesis and predicts a poor clinical prognosis^35^. However, Sirt1 mRNA levels are similar in HCC and non-tumor adjacent tissues, suggesting that Sirt1 overexpression was mediated by post-transcriptional mechanism in HCC^36^. Indeed, recent studies revealed that Sirt1 up-regulation could result from the downregulation of several microRNAs including miR34a, miR22 and miR133b in HCC^37–39^. In addition, MEK1 could suppress ubiquitination-dependent proteasomal degradation of Sirt1 to elevate its protein level^40^. Once up-regulated, Sirt1 can promote tumor progression by deacetylating multiple proteins in HCC cells. For example, Sirt1 can deacetylate YAP2 protein in HCC cells to activate YAP2/TEAD4-dependent transcription and induce cell growth^41^. In HCC, Sirt1 can also deacetylate and inactivate p53, leading to the transcriptional inactivation of tumor suppressor genes such as p21 and PUMA. Interestingly, deacetylated p53 also suppresses miR34a transcription, thus exerting a positive feedback loop between Sirt1 and miR34a in HCC^42^. We confirmed herein the overexpression of Sirt1 in HCC. Furthermore, knockdown of Sirt1 expression inhibits human HCC cell growth in vitro, and hepatocyte-conditional knockout of Sirt1 retards hepatocellular carcinogenesis in mice treated with DEN. Therefore, targeting Sirt1 might be a new strategy for the clinical management of HCC.

In summary, Sirt1 is up-regulated in HCC and promotes HCC development by directly deacetylating p62 to prevent its degradation. HCC development was comprised in mice with hepatocyte-specific Sirt1 knockout, which was rescued by overexpressing p62. Therefore, targeting Sirt1 or p62 is a reasonable strategy for the treatment of HCC.

## Supplementary information

Supplemental Figures and Tables

## Data Availability

The datasets and materials used and/or analyzed during the current study are available from the corresponding author on reasonable request.
