# Supplementary material for: Sirt1 deacetylates and stabilizes p62 to promote hepato-carcinogenesis
Source: Cell Death Dis. 2021 Apr 14;12(4):405. doi: 10.1038/s41419-021-03666-z (PMC8046979; doi:10.1038/s41419-021-03666-z)
Supplement: Supplementary file 1 — Supplemental Figures and Tables [file 41419_2021_3666_MOESM1_ESM.docx]

**Supplemental Figures and Tables**

**
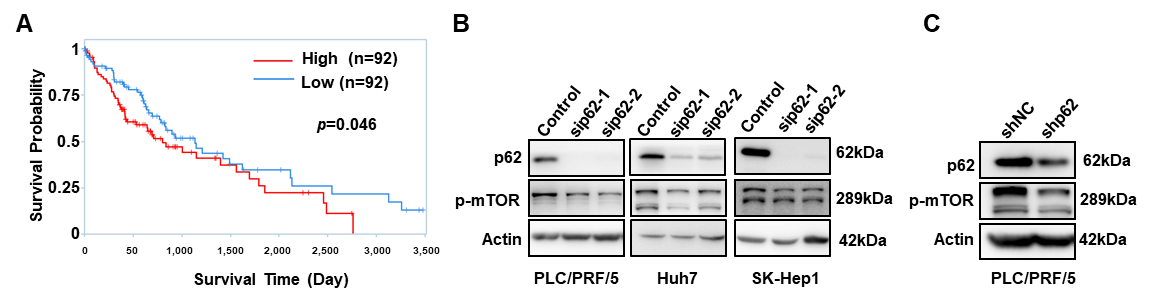
**

**Figure S1. High expression of p62/SQSTM1 promotes Hepatocellular carcinogenesis.**

(A) The impact of p62 protein expression on overall survival (OS) of HCC patients was analyzed on TCPA website (n=184, Cox-regression analysis; *p* < 0.05)

(B) The expression of p62 and the phosphorylation of mTORC1 in PLC/PRF/5, Huh7 and SK-Hep1 cells transient transfected with p62 or control siRNAs was detected by western blot.

(C) The expression of p62 and the phosphorylation of mTORC1 in PLC/PRF/5 cells stably transfected with p62 or control shRNA was analyzed via western blot.


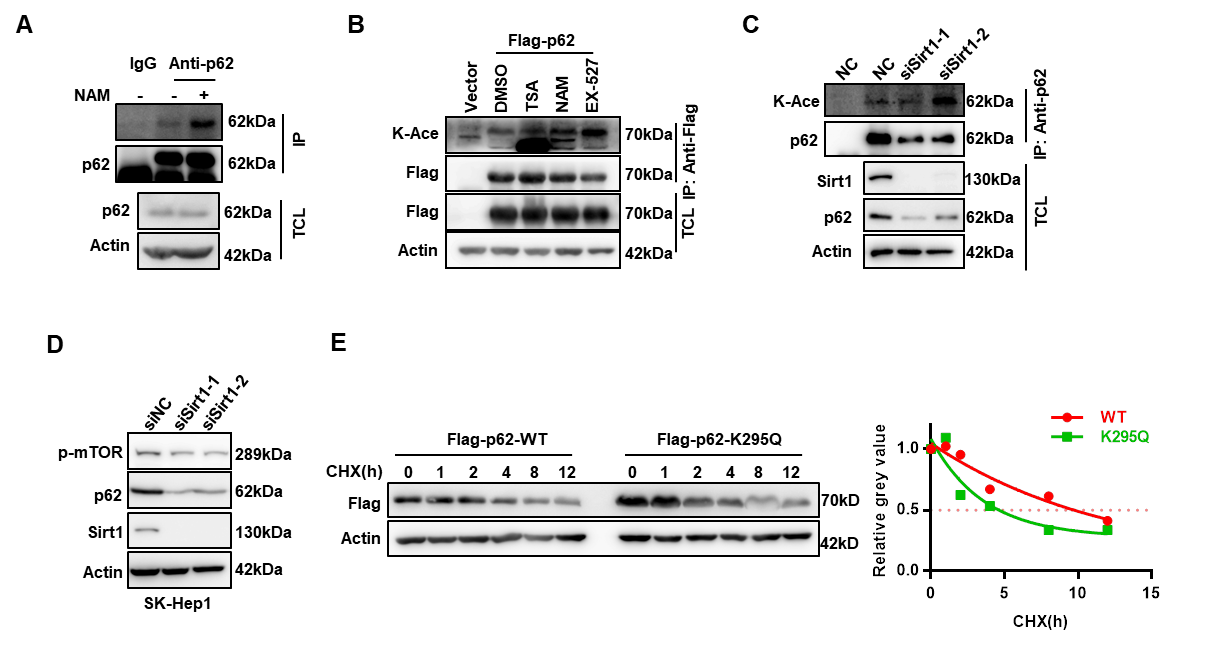


**Figure S2 Sirt1 deacetylases p62/SQSTM1.**

(A) Acetylation of endogenous p62 in PLC/PRF/5 cells treated with deacetylase inhibitors NAM (5mM, 6 hours) was detected by anti-p62 immunoprecipitation

(B) Acetylation of exogenous Flag-p62 in HEK293T cells treated with deacetylase inhibitors NAM (5mM, 6 hours), TSA (1μm, 12 hours) or Sirt1 inhibitor (EX-527, 25μM, 16 hours) was detected by immunoprecipitation with anti-Flag.

(C) Acetylation of endogenous p62 in PLC/PRF/5 cells treated with deacetylase inhibitors NAM (5mM, 6 hours) was detected by anti-p62 immunoprecipitation.

(D) The expression of p62 and the phosphorylation of mTOR (p-mTOR) in SK-Hep1 cells transient transfected with Sirt1 or control siRNAs was detected by western blot.

(E) Wild type (WT) of p62 or p62(K295Q) mutants were overexpressed in PLC/PRF/5 cells, followed cycloheximide (CHX) treatment with indicated time points, and the half-life of the WT and K295Q was detected via western blot, and the relative expression was measured by ImageJ software.


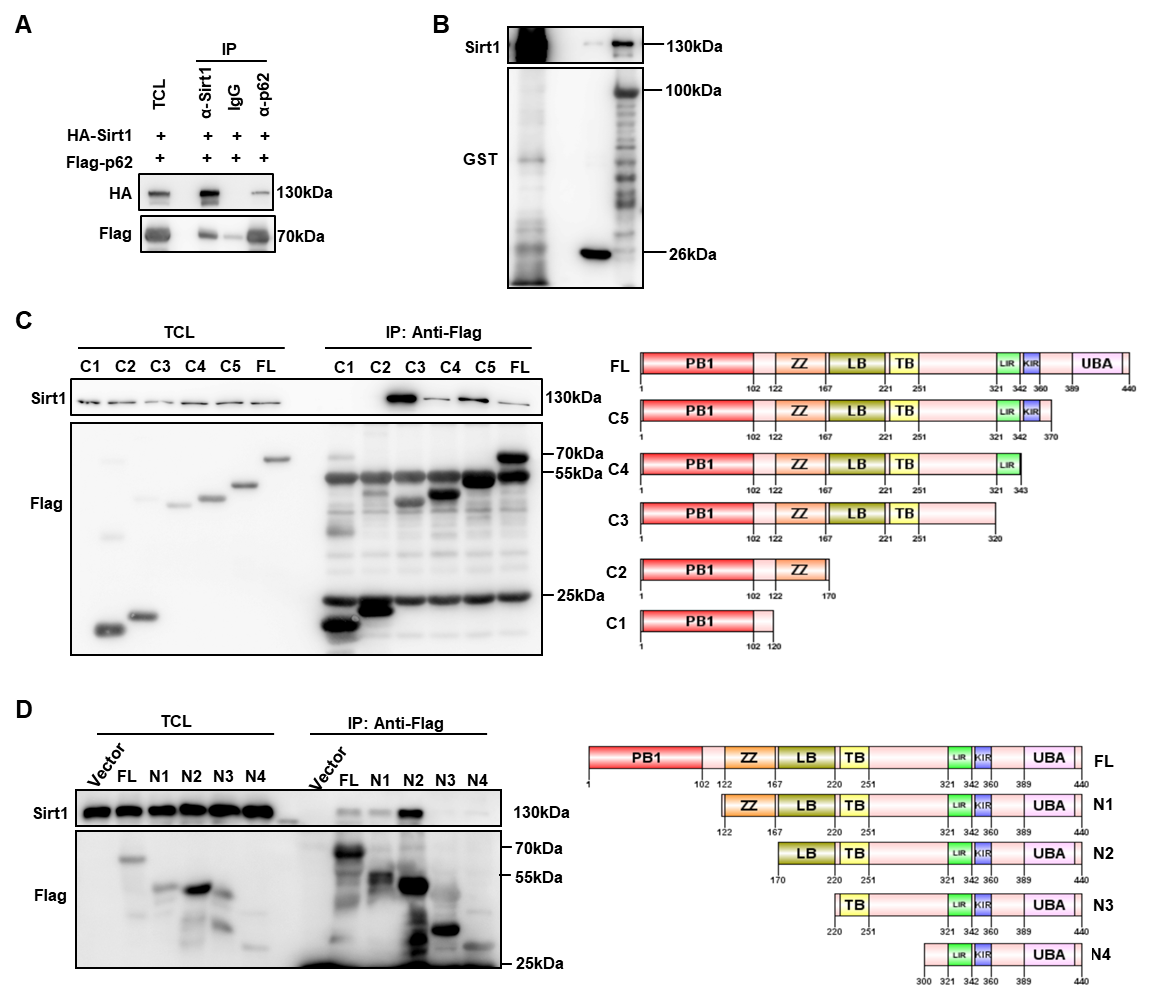


**Figure S3 Sirt1 directly interacts with p62/SQSTM1 via LB domain.**

(A) Co-immunoprecipitation (Co-IP) was performed to detect the interaction of exogenous Flag-p62 and HA-Sirt1 in HEK293T cells using anti-Sirt1 or anti-p62, blank IgG was used as the negative control.

(B) GST pull-down was performed with purified His-Sirt1 and GST-p62 or GST fusion proteins, and the interaction were detected via western blot using anti-Sirt1 or anti-GST.

(C) Co-precipitation of Flag-p62 and C terminal truncations (B) or N terminal truncations (D) with Sirt1 in HEK293T cells were analyzed.


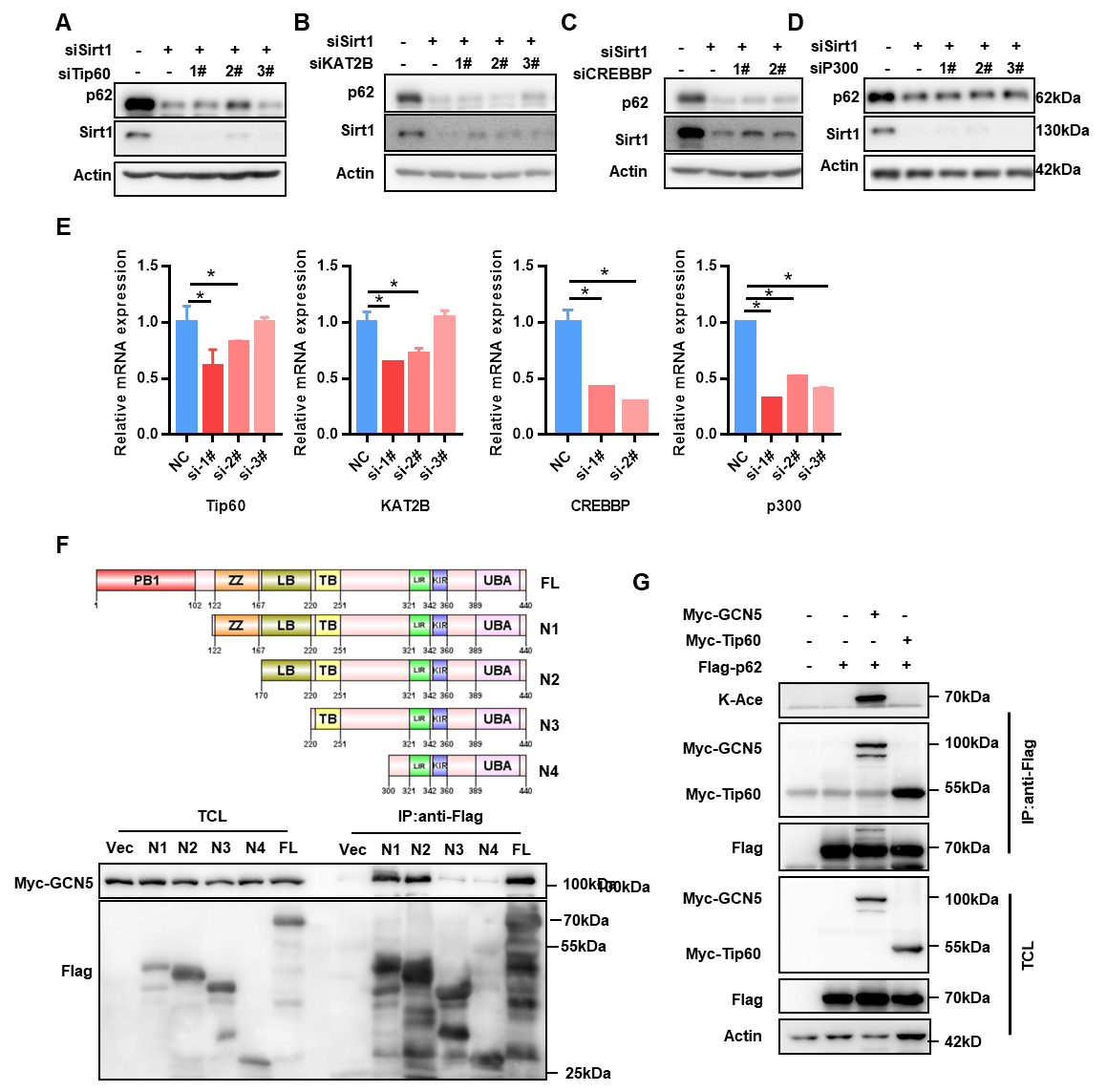


**Figure S4 GCN5 mediated p62 acetylation.**

(A) The expression of p62 in SK-Hep1 cells co-transfected with Tip60 and Sirt1 siRNA were analyzed by western blot.

(B) The expression of p62 in SK-Hep1 cells co-transfected with KAT2B and Sirt1 siRNA were analyzed by western blot.

(C) The expression of p62 in SK-Hep1 cells co-transfected with CREBBP and Sirt1 siRNA were analyzed by western blot.

(D) The expression of p62 in SK-Hep1 cells co-transfected with p300 siRNAs and Sirt1 siRNA were analyzed by western blot.

(E) The knockdown of Tip60, KAT2B, CREBBP or p300 in cells were validated by RT-PCR.

(F) Co-IP was performed to detect the interaction of Flag-p62 or its truncations with Myc-GCN5 in HEK293T cells with anti-Flag.

(G) Acetylation of Flag-p62 in HEK293T cells with overexpression of Myc-GCN5 or Myc-Tip60 were analyzed by anti-Flag immunoprecipitation, and probed with anti-K-ace.

**
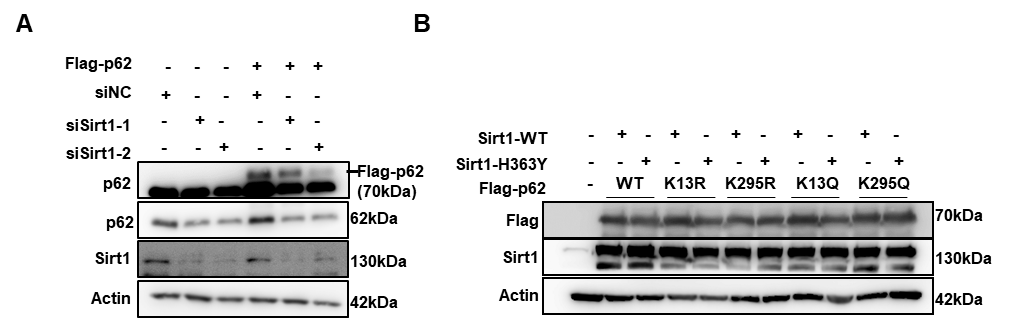
**

**Figure S5 Sirt1 stabilizes p62/SQSTM1 via inhibiting its acetylation.**

(A) Exogenous Flag-p62 protein expression in Huh7 cells with or without Sirt1 knockdown analyzed by western blot.

(B)The protein expression of exogenous p62 or its mutants with Sirt1-WT or Sirt1 enzyme inactive mutant overexpression via western blot.

**
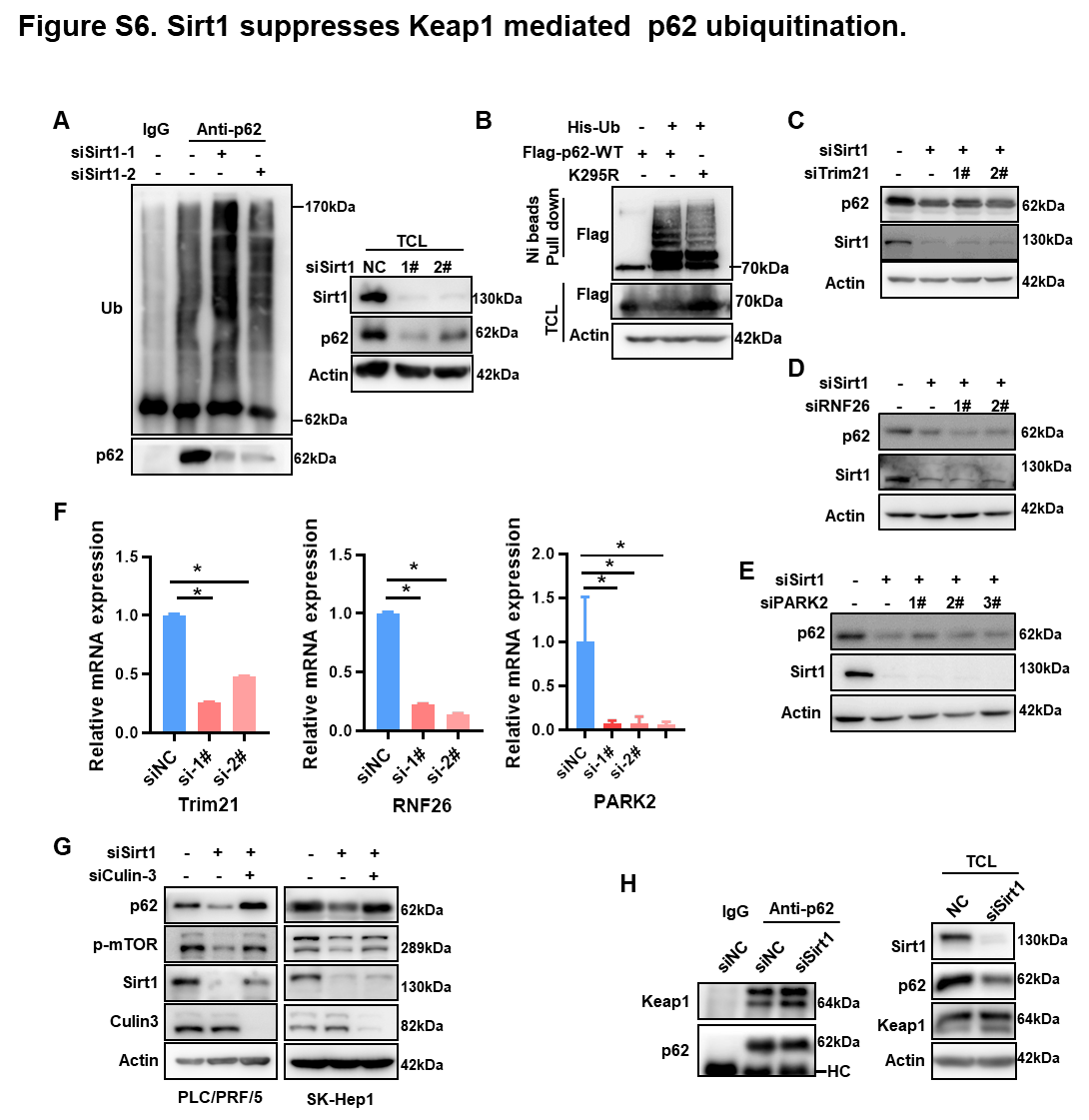
**

**Figure S6 Sirt1 protects p62 from Keap1-mediated ubiquitination dependent degradation.**

(A) Ubiquitylation of endogenous p62 in Sirt1 knockdown PLC/PRF/5 cells together with MG132 treatment was detected with anti-p62 precipitation, and probed with anti-Ub.

(B) Ubiquitylation of WT or K295R in HEK293T cells co-transfected with His-Ub or empty vector were detected with Ni-NTA beads pull down.

(C) p62 protein level in SK-Hep1 cells co-transfected with Trim21 siRNAs and Sirt1 siRNA were analyzed by western blot.

(D) p62 protein level in SK-Hep1 cells co-transfected with RNF26 siRNAs and Sirt1 siRNA were analyzed by western blot.

(E) p62 protein level in SK-Hep1 cells co-transfected with PARK2 siRNAs and Sirt1 siRNA were analyzed by western blot.

(F) Trim21, RNF26 or PARK2 knockdown in SK-Hep1 cells were validated by RT-PCR respectively (n=3, T-test, *p*<0.05).

(G) p62 protein level and the phosphorylation of mTORC1 in PLC/PRF/5 and SK-Hep1 cells co-transfected with Clu3 siRNAs and Sirt1 siRNA were analyzed by western blot.

(H) Interaction of p62 with Keap1 in PLC/PRF/5 after Sirt1 knockdown was analyzed by anti-p62 co-IP.

**
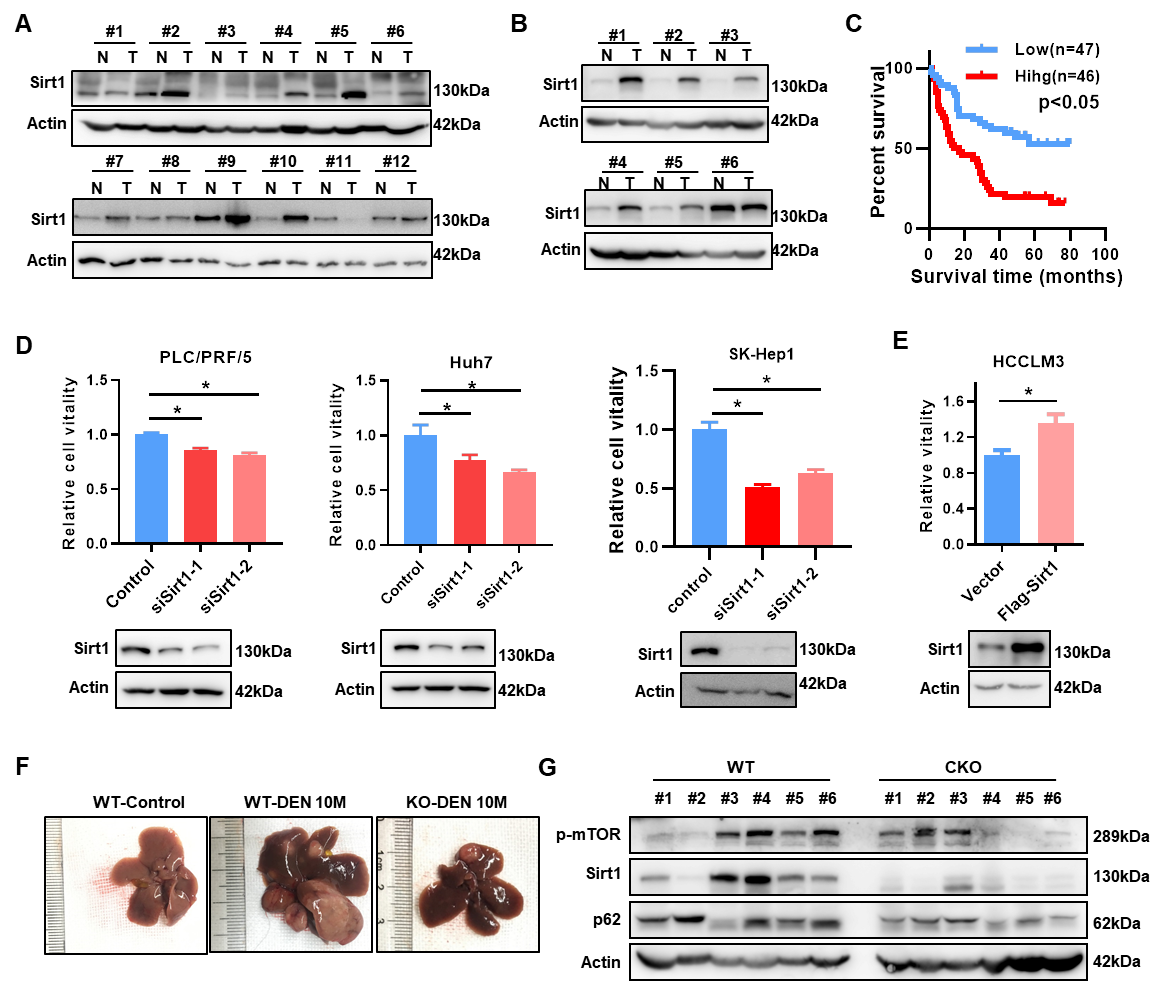
**

**Figure S7 Sirt1 promotes HCC tumorigenesis via stabilizing p62.**

(A)The protein expressions of Sirt1 in HCC and paired non-tumor tissues and mouse liver tissues mentioned in Figure1 were detected by western blot.

(B) The protein expressions of Sirt1 in DEN-induced mouse liver cancer and non-tumor tissues were detected by western blot.

(C)The impact of Sirt1 protein expression on overall survival (OS) of HCC patients was analyzed by Kaplan-Meier survival curve analysis (patients were grouped based on median Sirt1 expression; n=93, Cox-regression analysis; *p* < 0.05).

(D) Relative cell vitality of PLC/PRF/5, Huh7 and SK-Hep1 cells transient transfected with Sirt1 or negative control siRNAs (NC) were measured with MTS assay(n=10, T-test, *p*<0.05).

(E) Relative cell vitality of HCCLM3 cells transient transfected with Flag-Sirt1 or control vectors was measured with MTS assay(n=10, T-test, *p*<0.05).

(F)The representative liver images of three groups (wild type mice without DEN treatment, DEN treated wild type mice (Sirt1^fl/fl^, Alb-cre^-/-^, WT-DEN) and DEN treated Sirt1 hepatocyte specific knockout mice (Sirt1^fl/fl^, Alb-cre^+/+^, CKO-DEN) were showed respectively.

(G)The expressions of Sirt1, p62, and p62 downstream targets, p-mTOR were detected by western blot in liver tumors from WT-DEN or CKO-DEN groups.

**Table S1. Primer sequences used for Real-Time Polymerase chain reaction (PCR).**

| Human-Actin | F:ACTCTTCCAGCCTTCCTTCC  R:CGTCATACTCCTGCTTGCTG |
| --- | --- |
| Human-p62/SQSTM1 | F:AGAACGTTGGGGAGAGTGTG  R:GCGATCTTCCTCATCTGCTC |
| Mouse-Actin | F:TAGGCGGACTGTTACTGAGC  R:TGCTCCAACCAACTGCTGTC |
| Mouse-p62/SQSTM1 | F:CCCATCTACAGGTGAACTCC  R:CCTTCATCCGAGAAACCC |
| Human-Trim21 | F:TGTGCAGTCCACATCACTCTG  R:GGCCTCCTTTCCTGTCACAT |
| Human-RNF26 | F:CTGGTAGGCGTTGGGGTTAG  R:CCCCAAAGTCAGGTGCAGAT |
| Human-PARK2 | F:GCTGTGGGTTTGCCTTCTG  R:GGGCTGCGGACACTTCAT |
| Human-p300 | F:TGCGATCTGATGGATGGTCG  R:ACACAAGTCATAATCCTCACAGACA |
| Human-Tip60 | F:TCCTGGAGATCCTGATGGGG  R:TGAGGATGTACTGGCCCTTG |
| Human-KAT2B | F:AGCAGGTGAAGAGCCATCAAA  R:TCGCTGTAAGTCTGCCATGA |
| Human-CREBBP | F:CCAGAGACAAGCACTGGGAG  R:GTCGTAGTCCTCGCACACAG |

**Table S2. Primer sequences used for clone**

| K13R | F: CCACCCGGCCTGCTGGGATCAGAGCAG  R: CTACCTTCTGGGCAGGGAGGACGCGGC |
| --- | --- |
| K295R | F:GCCGCGTCCTCCCTGCCCAGAAGGTAG  R:CTGCTCTGATCCCAGCAGGCCGGGTGG |
| K13Q | F: GCCGCGTCCTCCTGGCCCAGAAGGTAG  R: CTACCTTCTGGGCCAGGAGGACGCGGC |
| K295Q | F: TTCCCACCCGGCTGGCTGGGATCAGAG  R: CTCTGATCCCAGCCAGCCGGGTGGGAA |
| p62-△LB | F1:TTCGGTACCCTCGAGTCTAGAATGGCGTCGCTCACCGTG  R1: TGATTCTGCCGTGGGGAATGCGAGCTTGGT  F2: CATTCCCCACGGCAGAATCAGCTTCTGG  GTCTTCGAAGGATCCACCGGTCAACGGCGGGGGATGCTT |
| C1(1-370) | F: ATTCTAGAATGGCGTCGCTCACCGT  R: ATACCGGTGGAGGGGTCCAGAGAGCT |
| C2(1-343) | F: ATTCTAGAATGGCGTCGCTCACCGT  R: ATACCGGTTGAAGACAGATGGGTCCAG |
| C3(1-330) | F: ATTCTAGAATGGCGTCGCTCACCGT  R: ATACCGGTGTTGCGGGGCGCCTCCT |
| C4(1-169) | F: ATTCTAGAATGGCGTCGCTCACCGT  R: GAACCGGTGGGGAATGCGAGCTTGG |
| C5(1-120) | F: ATTCTAGAATGGCGTCGCTCACCGT  R: ATACCGGT GTTGCGGGGCGCCTCCT |
| N1(121-440) | F: ATTCTAGAATGATGGTGCACCCCAATGTG  R: ATACCGGTCAACGGCGGGGGATCGTT |
| N2(170-440) | F: ATTCTAGAATGAGCCCCTTCGGGCACCTG  R: ATACCGGTCAACGGCGGGGGATCGTT |
| N3(220-440) | F: ATTCTAGAATGCCCACGGCAGAATCAGC  R: ATACCGGTCAACGGCGGGGGATCGTT |
| N4(300-440) | F: ATTCTAGAATGGTTGAGGGCGCCACGCAG  R: ATACCGGTCAACGGCGGGGGATCGTT |

**Table S3. siRNA sequences used for knockdown**

| **Name** | **Sequence** | **Supplier** |
| --- | --- | --- |
| siSQSTM1-1# | GUGACGAGGAAUUGACAAUTT  AUUGUCAAUUCCUCGUCACTT | Gene Pharma Company (Shanghai, China) |
| siSQSTM1-2# | GGAGUCGGAUAACUGUUCATT  UGAACAGUUAUCCGACUCCTT | Gene Pharma Company (Shanghai, China) |
| siSQSTM1-3# | CCAGACUACGACUUGUGUATT  UACACAAGUCGUAGUCUGGTT | Gene Pharma Company (Shanghai, China) |
| siSirt1-1# | GGAAAUAUAUCCUGGACAATT  UUGUCCAGGAUAUAUUUCCTT | Gene Pharma Company (Shanghai, China) |
| siSirt1-2# | GCAACUAUACCCAGAACAUTT  AUGUUCUGGGUAUAGUUGCTT | Gene Pharma Company (Shanghai, China) |
| siKeap1-1# | GGCGAAUGAUCACAGCAAUTT  AUUGCUGUGAUCAUUCGCCTT | Gene Pharma Company (Shanghai, China) |
| siKeap1-2# | GUCCUGCACAACUGUAUCUTT  AGAUACAGUUGUGCAGGACTT | Gene Pharma Company (Shanghai, China) |
| siKeap1-3# | GGGAGUACAUCUACAUGCATT  UGCAUGUAGAUGUACUCCCTT | Gene Pharma Company (Shanghai, China) |
| siTrim21-1# | GCAGAGCAUACCUGGAAAUTT  AUUUCCAGGUAUGCUCUGCTT | Gene Pharma Company (Shanghai, China) |
| siTrim21-2# | GAGGUCACAUGCCCUAUCUTT  AGAUAGGGCAUGUGACCUCTT | Gene Pharma Company (Shanghai, China) |
| siRNF26-1# | GAGAGGAUGUCAUGCGGCUTT | Gene Pharma Company (Shanghai, China) |
| siRNF26-2# | GCAGAUCAGAGGCAGAAGATT | Gene Pharma Company (Shanghai, China) |
| siPARK2-1# | GGAAGUCCAGCAGGUAGAUTT  AUCUACCUGCUGGACUUCCTT | Gene Pharma Company (Shanghai, China) |
| siPARK2-2# | GCUCCAUCACUUCAGGAUUTT  AAUCCUGAAGUGAUGGAGCTT | Gene Pharma Company (Shanghai, China) |
| siPARK2-3# | GGAAGCAGCCUCCAAAGAATT  UUCUUUGGAGGCUGCUUCCTT | Gene Pharma Company (Shanghai, China) |
| siGCN5-1# | CCCUGGAGAAGUUCUUCUATT  UAGAAGAACUUCUCCAGGGTT | Gene Pharma Company (Shanghai, China) |
| siGCN5-2# | CAGCCCUCCAUUUGAGAAATT  UUUCUCAAAUGGAGGGCUGTT | Gene Pharma Company (Shanghai, China) |
| siGCN5-3# | GCUCUACACAACCCUCAAATT  UUUGAGGGUUGUGUAGAGCTT | Gene Pharma Company (Shanghai, China) |
| siKAT5-1# | CCACAGGAACUCACCACAUTT  AUGUGGUGAGUUCCUGUGGTT | Gene Pharma Company (Shanghai, China) |
| siKAT5-2# | GCAAUGAGAUUUACCGCAATT  UUGCGGUAAAUCUCAUUGCTT | Gene Pharma Company (Shanghai, China) |
| siKAT5-3# | GCUUCCACAUCGUGGGCUATT  UAGCCCACGAUGUGGAAGCTT | Gene Pharma Company (Shanghai, China) |
| siKAT2B-1# | CCGCAUCAACUAUUGGCAUTT  AUGCCAAUAGUUGAUGCGGTT | Gene Pharma Company (Shanghai, China) |
| siKAT2B-2# | GGAAUUAAUCAACGAGGUUTT  AACCUCGUUGAUUAAUUCCTT | Gene Pharma Company (Shanghai, China) |
| siKAT2B-3# | GCAGGUGAAGAGCCAUCAATT  UUGAUGGCUCUUCACCUGCTT | Gene Pharma Company (Shanghai, China) |
| siEP300-1# | GGACUACCCUAUCAAGUAATT  UUACUUGAUAGGGUAGUCCTT | Gene Pharma Company (Shanghai, China) |
| siEP300-2# | CAUCACGGGUAUACAAAUATT  UAUUUGUAUACCCGUGAUGTT | Gene Pharma Company (Shanghai, China) |
| siEP300-3# | GAGGAGAGUAUACAUAUCUTT  AGAUAUGUAUACUCUCCUCTT | Gene Pharma Company (Shanghai, China) |
| siCREBBP-1# | GGCCUCCUCAAUAGUAACUTT  AGUUACUAUUGAGGAGGCCTT | Gene Pharma Company (Shanghai, China) |
| siCREBBP-2# | GGAGCCAUCUAGUGCAUAATT  UUAUGCACUAGAUGGCUCCTT | Gene Pharma Company (Shanghai, China) |
